# De-implementation of low-value home-based nursing care: an effect and process evaluation

**DOI:** 10.1186/s43058-025-00785-y

**Published:** 2025-10-01

**Authors:** Milou Cremers, Lisette Schoonhoven, Leti van Bodegom-Vos, Nienke Bleijenberg, Chantal Witsiers, Monique van Dijk, Erwin Ista

**Affiliations:** 1 Department of Internal Medicine, Section Nursing Science, Erasmus MC, University Medical Center Rotterdam, Dr. Molewaterplein 40, Rotterdam, 3015 GD the Netherlands; 2 Department of General Practice and Nursing Science, Julius Center for Health Sciences and Primary Care, University Medical Centre Utrecht, Universiteitsweg 100, Utrecht, 3584 CG the Netherlands; 3 School of Health Sciences, Faculty of Environmental and Life Sciences, University of Southampton, University Road, Southampton, SO17 1BJ UK; 4 Department of Biomedical Data Sciences, Leiden University Medical Center, Albinusdreef 2, Leiden, 2333 ZA the Netherlands; 5 Research Centre for Healthy and Sustainable Living, Faculty of Health Care, University of Applied Sciences Utrecht, Utrecht, 3584 CS the Netherlands; 6 Department of Neonatal and Pediatric Intensive Care, Division of Pediatric Intensive Care, Erasmus MC­-Sophia Children’s Hospital, University Medical Center Rotterdam, Dr. Molewaterplein 40, Rotterdam, 3015 GD the Netherlands

**Keywords:** De-implementation, Low-value care, Process evaluation, Nurses, Tailored strategies, Homecare

## Abstract

**Background:**

The demand for homecare is increasing, and reducing low-value care is essential for achieving sustainable healthcare. Low-value care refers to practices that are ineffective, inefficient, unwanted, or potentially harmful to the client. This study aimed to evaluate the effects of a tailored, multifaceted de­implementation strategy in reducing low-value home-based nursing care.

**Methods:**

A prospective, multicenter, convergent parallel mixed method design was employed, including a before-and-after study, using the Reach-Effectiveness-Adoption-Implementation-Maintenance (RE-AIM) framework. The effect of reducing low-value home-based nursing care was assessed from client records, focusing on the number of clients receiving care, minutes of care per week, frequency of visits per week, and clients no longer requiring care. The de-implementation process was evaluated qualitatively through individual interviews with de-implementation ambassadors, registered nurses, and nurse assistants, using Directed Qualitative Content Analysis. This approach served to interpret the effects of the deployment of de-implementation ambassadors and the strategies they implemented.

**Results:**

We observed a reduction in low-value home-based nursing care, with a decrease of 130 h per week in daily showering, bathing and/or dressing; 54 h per week in the assistance with compression stockings; and 8 h per week in changing bandages enabling clients to regain their independence. Important de-implementation strategies included involving clients and relatives in decision making, organizing informational meetings for homecare professionals, and fostering collaboration with other healthcare professionals. Factors that influenced adoption included providing reassurance and using a stepwise approach with clients and relatives. Homecare professionals noted that the de-implementation ambassadors were highly committed to reducing care. De-implementation ambassadors found their role to be intense, challenging, and exciting.

**Conclusions:**

This evaluation found that the deployment of de-implementation ambassadors, paired with additional de-implementation strategies, enhanced the reduction of low-value home-based nursing care. Providing reassurance and involving clients and their relatives were identified as beneficial for the de-implementation process.

**Supplementary Information:**

The online version contains supplementary material available at 10.1186/s43058-025-00785-y.

Contribution to the literature

• De-implementation ambassadors support teams to apply de-implementation strategies and reduce low-value home-based nursing care.
• It is possible to reduce low-value home-based nursing care while empowering clients to regain their independence.
• De-implementing low-value home-based nursing care not only frees up time and resources for more valued care, but also creates opportunities to accept new clients, contributing to a more accessible and sustainable homecare model.

## Background

As demand for homecare grows, reducing low-value care is essential for a more sustainable healthcare. Low-value care includes ineffective practices that cause more harm than benefit, inefficient care delivered suboptimally —such as being prolonged or overly frequent —and tasks clients could perform independently with care aids. It also encompasses unwanted care, where interventions, though effective, do not align with client preferences or conditions [1]. Such practices are common among nurses and nursing assistants in Dutch homecare organizations [2], including daily full-body bathing or assisting with tasks like applying compression stockings when clients could do so with care aids. With an expected 11% increase in nursing shortages in the Netherlands by 2027 (equivalent to 10,600 nurses) and an ageing population requiring more complex home-based care [3–5], reducing low-value care is increasingly urgent. Addressing this issue is crucial to ensuring value-based and effective care and maintaining future access to home nursing services.

De-implementation entails reducing, replacing or stopping practices that provide little to no benefit to clients. Reduction scales back the frequency or intensity of a practice while preserving its core purpose [6]. Replacement involves adopting a superior alternative, such as self-adhesive bandages (e.g. Coban™) instead of traditional bandaging. Stopping eliminates practices entirely when evidence advises against them, like discontinuing bladder irrigation to prevent catheter clogging [2, 7].

Successful de-implementation requires tailored strategies that address barriers and leverage facilitators for all stakeholders [6, 8, 9]. One key barrier is clients’ and relatives' high and unrealistic care expectations [10], which drive low-value care requests [8]. Conversely, clients’ motivation to regain independence serves as a facilitator [10]. Lack of knowledge about care aids e.g. stocking aids is another barrier. This can be overcome by training, which can help homecare professionals to develop their skills [10, 11]. Collaboration and team engagement are also critical, fostering ownership and clinical champions for de-implementation [9–13]. However, the most effective strategies for reducing low-value care remain unclear [14].

A promising approach is training of de-implementation ambassadors as champions [15]. Research highlights “team-directed learning” as an effective way to mobilize teams and improving compliance with recommended practices, such as good hand hygiene [16]. De-implementation ambassadors can drive change by serving as role models, applying de-implementation strategies, promoting behavior change, and ensuring sustainable reductions in low-value care within homecare teams [13, 16, 17]. This study aimed to evaluate whether de-implementation ambassadors, combined with tailored de-implementation strategies, effectively reduce low-value home-based nursing practices.

## Methods

### Design

**Fig. 1:**
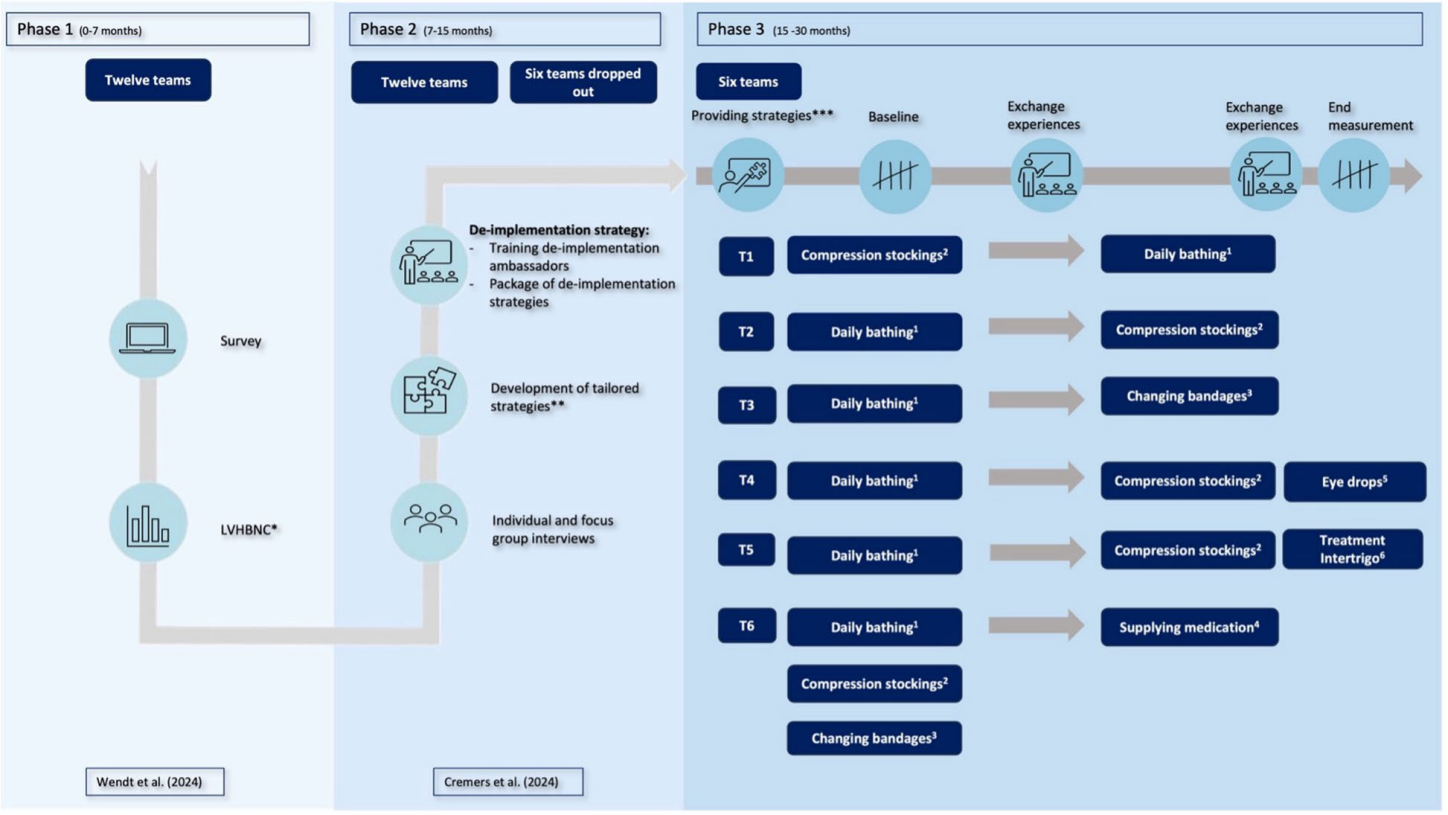
Overview of the complete phases of the DIMPLE study. Legend Fig. 1: *Low-value home-based nursing care (LVHBNC); **Systematic development of tailored strategies; ***Possible de-implementation strategies presented to de-implementation ambassadors; ^1^ Daily bathing/showering and dressing from head to toe; ^2^ Assisting with putting on/taking off compression stockings while the client can do this themselves with a care aid; ^3^ Choosing short-stretch bandages by default instead of using self-adhesive bandages (e.g. Coban™), UrgoK2, Farrow Wrap or Juxta Lite; ^4^ Assisting with medication while the client can do this him/herself with a device; ^5^ Assisting with administering eye drops while the client can do this him/herself with a care aid; ^6^ Applying zinc cream, powders or pastes when treating intertrigo

This study employed a prospective pragmatic multicenter before-after study, combined with a convergent parallel mixed-methods design. A qualitative evaluation explored the de-implementation process, focusing on the experiences of de-implementation ambassadors and homecare professionals in using the strategies and the factors influencing them. Quantitative data and qualitative data were collected, combined, compared, and integrated to reach an all-encompassing conclusion on effectively de-implementing low-value home-based nursing care practice [18]. The study followed the Reach, Effectiveness, Adoption, Implementation, and Maintenance (RE-AIM) framework, which guided both the effect evaluation (quantitative) and process evaluation (qualitative)[19]. Table 1 outlines the data collected during the process evaluation, and their alignment with the RE-AIM framework. Conducted as part of the DIMPLE-study (De-IMPLEmentation of low-value care in homecare nursing) (Fig. 1), this research adhered to the Standards for Reporting of Implementation studies (StaRI) checklist for comprehensive and transparent reporting [20].

**Table 1:** RE-AIM evaluation plan on the reduction of low-value home-based nursing care

RE-AIM domain	Definition	RE-AIM questions	Measure / Indicator	Data Source	
Effect evaluation: What was the effect of using de-implementation ambassadors and de-implementation strategies on the reduction of low-value home-based nursing care practices				Client records	
**Reach**	The number and proportion of clients receiving the targeted low-value practices (e.g. assistance with showering, dressing or compression stockings) who were assessed for de-implementation opportunities by the ambassador and homecare nurses. This includes both existing and new clients who started receiving care during the study period	How many clients received the chosen nursing care practices, were approached to reduce these care practices because of the low-value, and care was reduced and/or left care	% of Number of clients	X	
**Effectiveness**	The proportion of clients for whom the low-value care was successfully reduced or discontinued	In how many clients could nursing care practices reduced or de-implemented/discontinued?	Number and proportion of LVHBNC addressed; Changes in frequency and documented minutes per week spent on LV-HBNC practices before and after the implementation of strategies; The number and proportion of clients who no longer required the care (e.g., due to regained independence)	X	
**Process evaluation:** What were the experiences of using de-implementation ambassadors mobilizing teams to apply tailored de-implementation strategies on the reduced low-value home-based nursing care practices				Field notes	Interviews
**Reach**	Which stakeholders were relevant to reach, and which strategies were used for reaching these stakeholders?	Who were relevant and accomplished to reach by de-implementation ambassadors for reducing low-value home-based nursing care and which strategies were used to reach them?	Overview of strategies Overview of influencing factors	X	X
**Adoption**	The number of de-implementation ambassadors participating in reducing low-value home-based nursing care	How many de-implementation ambassadors were included, went through with de-implementing or dropped out during the process and what reasons were there for dropping out?	Number of de-implementation ambassadors	X	
	The number of teams that are willing to initiate the DIMPLE program	The willingness of homecare teams to participate in the reduction of low-value home-based nursing care	Number of homecare teams	X	
	Homecare professionals’ and de-implementation ambassadors’ experiences with the role of the de-implementation ambassadors	What were the experiences with the de-implementation ambassador and what experienced de-implementation ambassadors in their role as?	Overview of influencing factors	X	X
	The willingness of the stakeholders to participate in the reduction of low-value home-based nursing care	Which factors influence the willingness to participate in the reduction of low-value home-based nursing care?	Overview of influencing factors	X	X
**(de)- Implementation**	Homecare professionals’ experiences with the de-implementation process, strategies used, and influencing factors on reducing low-value home-based nursing care practices	What were the experiences with the used strategies for the reduction of low-value home-based nursing care?	Overview of strategies Overview of influencing factors	X	X
		Which factors were experienced by homecare professionals on reducing low-value home-based nursing care practices?	Overview of influencing factors	X	X
**Maintenance**	The structural changes in work care process after the used strategies and future aspects for reducing low-value home-based nursing care	which structural changes in work care process were made after using the strategies?	Overview of influencing factors	X	X
		Which future aspects were mentioned for reducing low-value home-based nursing care?	Overview of influencing factors	X	X

*LVHBNC* Low-value home-based nursing care

### Setting and participants

Twelve teams from three different homecare organizations in the south-western (*n* = 1) and mid-central (*n* = 2) regions of the Netherlands participated in the study. The study population included nursing assistants (levels 2 and 3) and registered nurses (levels 4 and 6). All organizations operated in urban areas, comprising 8 to 100 teams and employing between 95 and 1,500 employees.

Each homecare team consisted of 7 to 20 professionals with varying levels of training. Nursing assistants focused on daily activities and low complexity nursing tasks, such as bathing and dressing clients. Registered nurses provided more complex care, coordinated services, and – at level 6 – conducted needs assessments (Supplementary materials 1). These assessments, performed at the start of care provision, involved collaboration with clients and their networks to determine required care. The goal was to strengthen clients’ ability to care for themselves and to support, promote, and sustain their ability to perform essential activities [21, 22]. Required care in Dutch home healthcare is documented in an individual care plan, which specifies the type of care and the number of minutes allocated for homecare professionals to perform this care. These care minutes are used for reimbursement by health insurance companies. If the required care changes – either increasing or decreasing – the care plan is updated accordingly, including the number of billable minutes.

#### Recruitment, inclusion and exclusion criteria

Within each team, a registered nurse or nursing assistant was voluntary appointed as a de-implementation ambassador, leading efforts to reduce low-value home-based nursing care and support applying de-implementation strategies. In addition, ambassadors and team members were invited for participation in the qualitative evaluation that explored the de-implementation process. Ambassadors and team members were eligible for interviews if they had worked within the team for at least one year. This ensured they were familiar with procedures prior to de-implementation and could observe changes after the process. Nursing students without a healthcare degree and self-employed workers were excluded, as they typically worked in teams for less than a year or were only temporarily employed, limiting their knowledge of the de-implementation procedures.

### DIMPLE program

In the DIMPLE (De-IMPLEmentation of low-value care in homecare nursing) program, we aimed to actively reduce, replace or stop low-value home-based nursing care practices. Examples of high-prevalence, low-value home-based nursing care practices derived from Dutch national nursing guidelines and surveyed in a recent questionnaire study include 'washing the client with water and soap by default', 'washing the client from head to toe daily' and 'applying zinc cream, powders or pastes when treating intertrigo' (see Fig. 1) [2]. At the same time, the program aimed to strengthen clients’ self-reliance and their ability to perform essential daily activities [21, 22]. Teams were instructed not to de-implement care in cases where it was deemed necessary e.g. for frail elderly clients. In fact, appropriate care was provided in those clients. Clients were reassured that care would be reinstated if the change proved unsuccessful. De-implementation ambassadors also conducted evaluation interviews with clients to monitor the process of reducing or stopping low-value care.

### Tailored de-implementation strategies (DIMPLE-strategy)

Implementation mapping was used to develop the multifaceted de-implementation strategies [23]. These strategies were based on identified barriers and facilitators related to the top ten low-value home-based nursing care practices [2, 10].

First, all determinants were categorized as either generic (applicable to all low-value care practices) or specific to a particular practice. During two meetings, the research team (EI, LvB, LS and MC) further classified each determinant as pertaining client-related, team-related, or organizational in nature. Determinants were then prioritized based on their perceived importance for enabling de-implementation and the frequency with which they were mentioned during data collection. The criteria for prioritization were as follows:

- High priority: essential for enabling de-implementation and mentioned very frequently,
- Moderate priority: influential and mentioned frequently,
- Low priority: mentioned only once or perceived to have a limited impact.

All steps in this process were discussed in the team and consensus was reached at each stage.

**Fig. 2:**
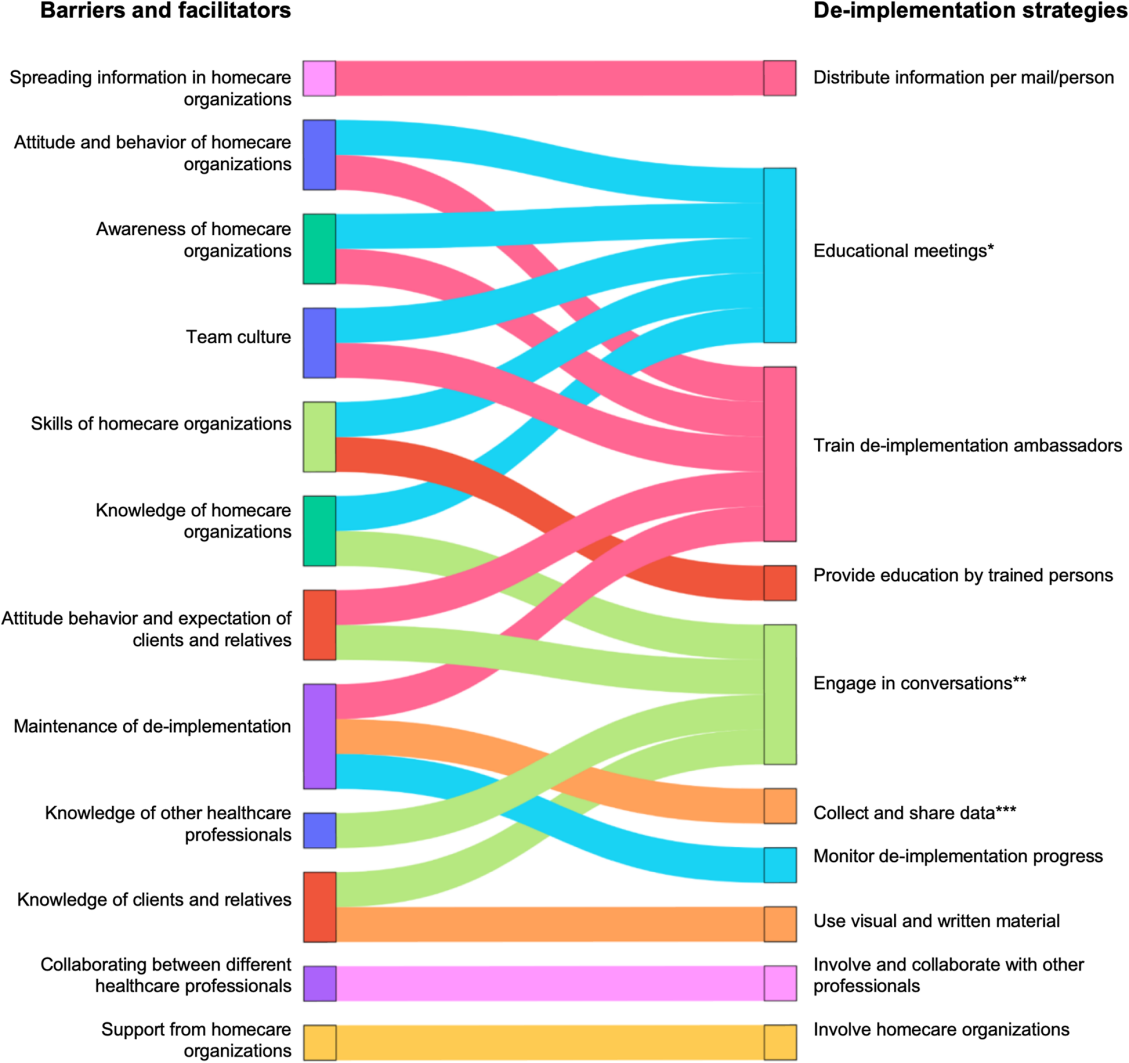
Used DIMPLE strategies mentioned by de-implementation ambassadors during coaching meetings and individual interview for de-implementing low-value home-based nursing care practices linked to the identified barriers and facilitators (Cremers et al. 2024). Legend Fig. 2: * Educational meeting to increase knowledge, create awareness, share experiences, share feedback, discuss pros, cons, and importance of reducing care, ** Engage in conversation with the stakeholders, such as clients and relatives, individuals in the homecare team, or other healthcare professionals (e.g., general practitioners or occupational therapist), *** Share data with homecare teams and homecare organisations

While we recognized that determinants might differ between low-value practices, we opted for an overarching analysis to ensure feasibility and to allow identification of cross-cutting determinants that could be addressed through common strategies. Next, the resulting high- and moderate priority determinants were matched with behavior change methods [24] and subsequently aligned with implementation strategies from an established taxonomy [17]. Lastly, these de-implementation strategies were operationalized and reviewed for clarity and consistency by an expert panel of five homecare professionals [25]. A total of 15 strategies were developed across four levels: homecare teams, client and relatives, other healthcare professionals, and homecare organizations Fig. 2 (Supplementary materials 3). One of the core strategies was the de-implementation ambassador, who served as clinical champion to support, promote and guide the de-implementation while addressing resistance if needed [17]. De-implementation ambassadors participated in a training and coaching program to understand the de-implementation process, apply its steps, and coach their teams in reducing low-value care. The training included six sessions before implementation and two sessions during the process. More detailed information about the training and coaching program is provided in Supplementary materials 2.

### Study procedure and data collection

Each de-implementation ambassador, together with their team, selected one or more low-value care practices to address, as shown in Fig. 1. The selection was based on how many clients received the care practice and how often this was considered low value care. These practices were categorized as: 1) care practices replaced by implementing a care aid, device or different techniques, and 2) care practices reduced in intensity or frequency.

#### Quantitative data collection—Client records

Reach was defined as the proportion of clients receiving the targeted low-value practices (e.g. assistance with showering, dressing or compression stockings) who were assessed for de-implementation opportunities by the ambassador and the nurse. This includes both existing and new clients. Effectiveness was defined as the proportion of clients for whom the low-value care was successfully reduced or discontinued (Table 1). To evaluate Reach and Effects of de-implementation of low-value care practices each ambassador compiled a list of clients receiving the targeted care practices. In collaboration with the nurse, the ambassador assessed whether the care was appropriate for each client and whether it could be reduced or de-implemented/discontinued. The ambassador continuously updated the list to include new clients. For each client, the de-implementation ambassador extracted data directly from the care plan at baseline and again after nine months. This included the number of minutes allocated per week the low-value care practice, frequency of visits per week related to that care, and the low-value care episodes. For new clients, the de-implementation ambassador extracted the data at the start of the provision and again at the time of data collection.

#### Qualitative data collection—Experiences of the de-implementation ambassadors and tailored strategies

##### Field notes

After coaching meetings, the researcher (MC) documented relevant observations in a logbook, including strategies used, effects on reducing low-value care, and influencing factors. This logbook also tracked team dropouts and the reasons for withdrawal.

##### Individual interviews

After nine months, to evaluate the implementation process we performed semi-structured interviews (MC or CW), separately with de-implementation ambassadors and two team members per team with differing educational levels and involvement in low-value care. This approach ensured a diversity of experiences regarding the de-implementation of low-value care practices. Interviews took place at the homecare organization’s workplace, with Microsoft Teams used as an alternative when in-person meetings were not feasible. Before the interviews, participants completed a demographic questionnaire, gathering information such as gender, role, and years of homecare nursing experience. A structured interview guide, based on the RE-AIM framework, was tailored to each participant group. The guide for de-implementation ambassadors included questions about the training and coaching they had received, while the guide for team members focused on the ambassadors’ role in the de-implementation process (Supplementary materials 4). An independent researcher (CW) uninvolved in the de-implementation process conducted interviews with de-implementation ambassadors, ensuring they could freely discuss their training and coaching experiences. All interviews were audio-recorded and transcribed verbatim. Participants received a summary of their transcript and had one week to provide feedback. If no response was received, agreement with the summary was assumed.

### Data analysis

Descriptive statistics were used to analyze the quantitative data, including participant characteristics, the number of minutes of care provided per week before and after de-implementation, the visit frequency per week before and after de-implementation, and reasons for care discontinuation. Normally distributed data were presented as mean (standard deviation), while non-normally distributed data were reported as median (Interquartile range) values. There were no missing data, and all analyses were conducted using IBM SPSS Statistics for Windows, Version 28.0. Armonk, NY: IBM Corp.

Fieldnotes and individual interviews were analyzed using Directed Qualitative Content Analysis [26]. Initial coding was guided by the RE-AIM framework and previously identified barriers and facilitators. A deductive approach was used to code relevant data segments. For data that did not fit predetermined codes, an inductive approach was applied.

The first four interviews were independently analyzed by MC and CW. Codes were compared, and any discrepancies were discussed and resolved collaboratively. The remaining transcripts were coded independently by either MC or CW and then reviewed by the other. Any discrepancies were discussed, reassessed, and resolved by consensus. Data collection continued until data saturation was reached, ensuring no new information. The qualitative data provided context and explanation for the quantitative findings on reducing low-value home-based nursing care.

## Results

### Effect evaluation—reducing low- value home-based nursing care

#### Reach

During the study period, a total 252 clients were screened for reducing low-value home-based nursing. Initially, 152 of the 252 clients (60.3%) required assistance with (daily) showering, bathing and/or dressing. Regarding compression stockings, 89 clients (35.3%) initially received assistance. Eleven clients (4.4%) required care assistance for changing bandages.

#### Effectiveness

Daily showering, bathing and/or dressing was reduced for 37 clients (24.3%), and completely discontinued for 24 clients (15.8%). Among those for whom care was discontinued, ten clients regained independence, while 9 clients experienced a decline in health and required increased support, resulting in transition to a nursing home (Table 2). These clients, along with those who have passed away and been transferred to other care organizations, were excluded from the calculation of reduced care. Because in some cases, care was not reduced or discontinued because clients were considered too frail or their health status was already deteriorating. Several of these clients were later admitted to nursing homes reflecting an expected transition in care needs rather than an unintended consequence of de-implementation (Table 2).

**Table 2:** Cases of clients receiving homebased nursing care practices

	Potentially Low value home-based nursing care practice		
	Daily showering, bathing and/or dressing *(6 homecare teams)*	Compression stockings *(4 homecare teams)*	Changing bandages *(2 homecare teams)*
Number of clients receiving care^a^	*N* = 152	*N* = 89	*N* = 11
Appropriate care, n (%)	84 (55.3)	60 (67.4)	2 (18.2)
Increased care^b^, n (%)	7 (4.6)	2 (2.3)	-
Care reduced^c^, n (%)	37 (24.3)	12 (13.5)	7 (63.6)
Care stopped^d^, n (%)	24 (15.8)	15 (16.9)	2 (18.2)
Reasons for stopped care: - Nursing home - Regain independence - Passed away - Other organization	9 (37.5%) 10 (41.6%) 3 (12.5%) 2 (8.3%)	2 (13.3%) 10 (66.7%) - 3 (20%)	- 1 (50%) 1 (50%) -

*n* number of clients

^a^% and number of clients receiving one or more of three targeted care practices, both value or low value care in the participating teams

^b^% and number of clients who needed more care due to deterioration in healthcare status

^c^% and number of clients with reduced low-value care

^d^% and number of clients for whom care stopped

Care regarding compression stockings was reduced for 27 of these clients (30.4%), and 15 clients (16.9%) no longer required support, of whom ten regained independence (Table 2). In addition, switching to self-adhesive bandages led to care reductions for 9 (81.8%) out of 11 clients (Table 2).

**Fig. 3:**
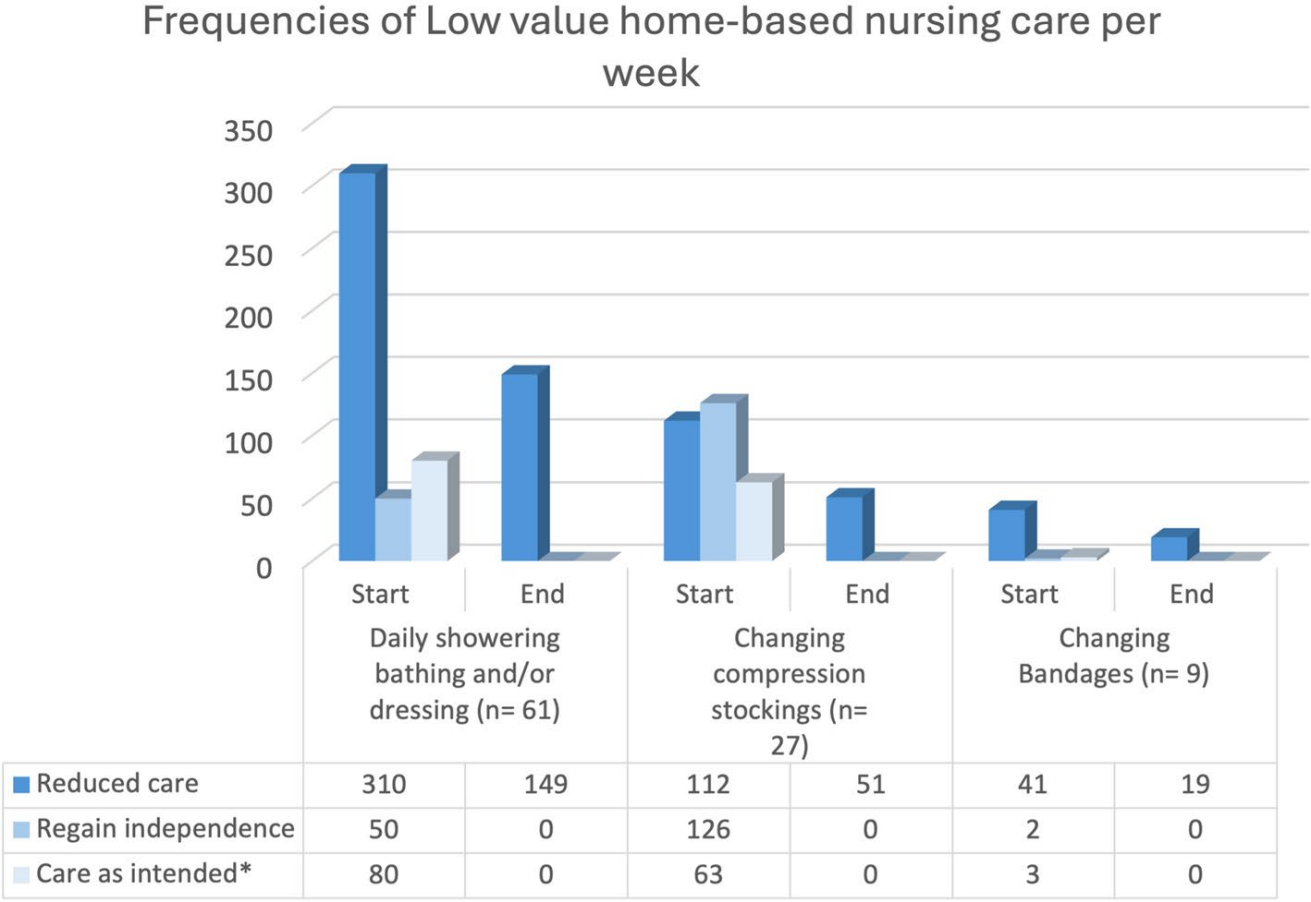
Frequencies of Low value home-based nursing care per week. Legend Fig. 3: All clients whose care was reduced or stopped included in Table 2 were included. * Care as intended; this means the client received this type of care and we did not start and/or stop reducing low-value care practices because clients were transferred to nursing home, changed organizations or passed away

**Fig. 4:**
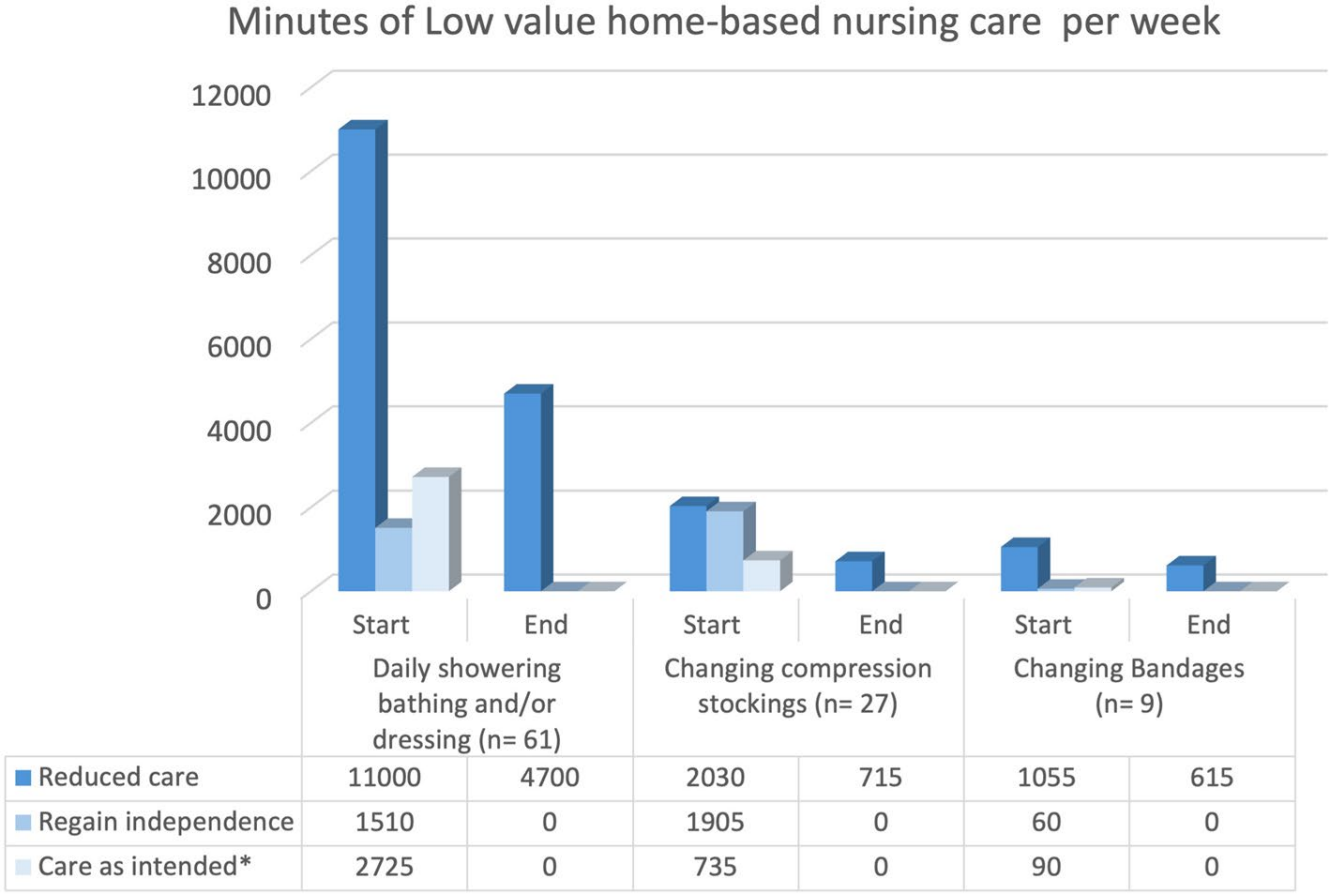
Minutes of Low value home-based nursing care per week. Legend Fig. 4: All clients whose care was reduced or stopped included in Table 2 were included. * Care as intended; this means the client received this type of care and we did not start and/or stop reducing low-value care practices because clients were transferred to nursing home, changed organizations or passed away

A decrease was measured in weekly home visits for clients who required assistance with (daily) showering, bathing and/or dressing from 360 at baseline to 149 at the end of the process (Fig. 3). This corresponded to an overall time reduction of 7,810 min per week (from 12,510 to 4,700) (Fig. 4).

Regarding compression stockings, weekly visit frequency for compression stocking assistance dropped from 238 to 51, and total care time decreased from 3,935 min to 715 min per week (Figs. 2 and 3). Switching to self-adhesive bandages led to reductions for 9 out of 11 clients (Table 2), cutting weekly visits from 43 to 19 and total care time from 1,115 min to 615 min per week (Figs. 3 and 4).

### Process evaluation

A total of 17 participants were interviewed, including six de-implementation ambassadors and 11 team members from six different teams across three homecare organizations. The majority of interviewees were women (82.4%), working as a registered nurse level 4 (35.3%) or level 6 (35.3%) (Table 3). Interview durations ranged from 17 to 51 min. Interviews were conducted in person, except for one with a de-implementation ambassador, which was held online via Teams and took place between February 2024 and July 2024.

**Table 3:** Characteristics of participants (*n* = 17)

	N	%
**Gender**		
Female	14	82.4
Male	3	17.6
**Age**		
20 – 29 years	4	23.5
30 – 39 years	2	11.8
40 – 49 years	3	17.6
50 – 59 years	6	35.3
< 60 years	2	11.8
**Profession**		
Health and Welfare assistant (Level 2)	1	5.9
Certified Nursing assistant (Level 3)	4	23.5
Registered Nurse (Level 4)^a^	6	35.3
Registered Nurse (Level 6)^a^	6	35.3
**De-implementation ambassador or team member**		
De-implementation ambassador	6	35.3
Team member	11	64.7
**Experience in home healthcare**		
< 5 years	3	17.6
5 – 14 years	5	29.4
15 – 24 years	5	29.4
25 – 34 years	2	11.8
< 35 years	2	11.8

^a^NQF Level 4 is vocationally trained registered nurses and level 6 is bachelor trained registered nurses. Level 6 has expanded tasks compared to level 4, these can be found in Supplementary materials 1

#### Reach

##### Reaching relevant stakeholders

All de-implementation ambassadors emphasized the importance of engaging team members, clients, relatives, and other professionals such as general practitioners (GPs) and occupational therapist (OTs). Homecare professionals were reached through educational meetings and one-on-one conversations led by de-implementation ambassadors (Fig. 2). These meetings focused on increasing awareness, for example, by highlighting the importance of self-care. Both homecare professionals and de-implementation ambassadors informed current clients and their relatives about reducing low-value care through direct conversations. For new clients, these conversations took place during needs assessments before care was provided. During these conversations, expectations were clarified, agreements were made, and care goals were set (Fig. 2).

*“I involve relatives immediately in the conversation, especially if they are the client’s children or a spouse. You must involve them in conversation when you are going to reduce care. They were very understanding” participant 8.*

De-implementation ambassadors used various strategies to engage other professionals, such as GPs, primarily through one-on-one conversations. These conversations served not only to communicate new approaches but also to introduce themselves and their role in the de-implementation process (Fig. 2). However, de-implementation ambassadors faced challenges in reaching GPs due to the large number of GPs operating within the homecare teams’ service area (Table 4).

**Table 4:** Influencing factors for the de-implementation process

**Factors on …**	**Homecare professionals experienced these factors as positively on the de-implementation process …**	**Homecare professionals experienced these drivers as negatively on the de-implementation process …**
Reaching (new) clients	- New clients; communicating which care will be provided; establishing expectations; reaching explicit agreements; Immediately evaluating whether clients can do it with a care aid - Team members or de- implementation ambassador engage conversations with clients considered reducing LVHBNC	- Client health conditions (for example dementia, loss of strength, physically challenged, rheumatism, old age, obesity, incontinence, last stages of life, wounds; psychological challenges, stroke, asthma etc.)
Reaching homecare teams	- Communicating new agreements for conducting needs assessment with other district nurses on the team - Interaction between de-implementation ambassador and team members - Team members were not against it, but also not strongly in favor of the de-implementation	- Few team members go their own way and miss new agreements - Unaware that they will reduce/stop LVHBNC as a team
Reaching other professionals	- Good communication and talking regularly makes it easier to inform and address LVHBNC together, for example with GPs or OTs - Making yourself known as a point of contact for other disciplines - Communicated new approach towards other professionals	- GPs ask to provide care that is considered LVHBNC, because a client asks for it; and are not informed on the various care aids - Many different disciplines and individuals to reach and no regular meeting planned, for example multiple GPs need to be reached separately
Reaching other healthcare organization		- Hospital settings are experienced as difficult and complex to reach because of many departments, various specialisms, and many various professionals - Care requests from hospitals and rehabilitation centers for providing care that is considered LVHBNC, and do not always correspond to the care needed - Experience that in hospitals and rehabilitation centers do not consider potential care aids for providing care
Adoption of clients and relatives	- Establishing expectations ensures that clients are open to reducing care - Benefits of selfcare, such as less rotations in visits of homecare professionals - Clients motivated to be independent - Provide insight for client and relatives into expenses made versus paying for health insurance - Examine the cause of resistance and address that; for example, safety, then optimize safety - Offer reassurance; care can be restarted if needed	- Clients feel they are entitled to receive care because they pay for it, - Client and relatives think the contribution for care aids is too expensive - Client are of the opinion that it is the homecare professional's job - Clients are afraid that if they reduce care, they will no longer be able to receive care if needed - Relatives consider it a reassuring feeling that homecare professionals visit daily
Adoption of the homecare team	- Registered nurses’ level 6 more informed on the topic compared to nursing assistants, - Increased knowledge about possible care aid that can be used - Create awareness about the importance of motivating self-care, that time is occupied for new clients and that it is necessary to keep care accessible in the future	- Working by habit, and are not aware of providing LVHBNC - Previous mindset in which the client is king and is washed from head to toe - Feeling of being resented when you reduce or do not perform care - High work pressure; too busy to read care plan, more likely to take care from clients, due to workload too tired to address de-implementation of LVHBNC, usage of care aid lower priority due to work pressure
Adoption of the role of ambassadors	- De-implementation ambassador found it exciting and challenging to be involved in reducing care - Recommended to have a colleague from the team involved in the project, so that they shared burdens and had opportunities to brainstorm together - De-implementation ambassadors encourage, compliment and motivate others to take steps in reducing LVHBNC, and function as a point of contact in case of resistance or problems - Brainstorming with other de-implementation ambassadors, sharing results, coaching meetings with the researcher, practicing conversation with volunteers and discussing the de-implementation process - Having to invest a lot of time in the beginning to get through client database,	- Plan of action was difficult to create in the beginning - Registered nurse level 6 already has a lot of tasks and responsibilities. It was difficult to also perform the role of de-implementation ambassador
Adoption between professionals	- Communicate to GPs; what care is provided, and the importance of LVHBNC reduction - GPs open to the new approach and ask advice from homecare professionals - Engaging OTs to help with learning care aids and requesting advice on care aids	
De-implementation for clients and relatives	- Clients are satisfied to regain their independence, organize their own day - Often clients get compression stockings on or off, but not both - Use of visual material and a care aid to practice helped client towards self-care - Use a step-by- step approach; Establish expectations, set goals and then evaluate them - Involving relatives and clients and allowing them to decide, for example, in choosing which days care is provided	- Immediately reducing care without steps, relatives called that this was way too fast - Clients return to care, have not been adherent after reducing therapy; more time needed
De-implementation in the homecare teams	- New care request; district nurses immediately use possible aids, making agreements, setting goals, expressing expectations and limits - Allowing clients time to perform care themselves and saying ‘no’ when the client asks to take over the care - In small groups, reduce together with clients, easier to communicate steps to undertake when reducing - Sharing achievement of goals even small ones, for example share results during team meetings. Seeing results makes one follow - Provide feedback and address each other when LVHBNC is performed - Team culture; discuss care requests with each other, motivate each other, encourage each other to continue to reduce care that is LVHBNC, work in collaboration with each other - A team culture in which care provided is critically evaluated, new changes get embraced, and mindset what the client can do himself, he does himself - Result of regaining independent feels rewarding	- Reducing bathing and showering are more difficult conversations, than when you start using a care aid - Results of reducing LVHBNC were not shared - Not aware of who the de-implementation ambassador is in the team - Team culture; Our homecare team does their own thing and if clients have been in care for a long time, care plans are not reviewed - Team culture in which colleagues disagree on agreements - Team members identify LVHBNC but then do not act on it - Homecare professionals have difficulty saying ‘No’ to clients and relatives - Due to workload sometimes accidentally still new LVHBNC accepted or reducing LVHCNC became less of a priority
De-implementation on the ambassadors	- Brainstorming with other de-implementation ambassadors, sharing results, coaching meetings with the researcher, practicing conversation with volunteers and discussing the de-implementation process - DIMPLE step-by-step plan that assists in reducing LVHBNC - Homecare professional mentioned that de-implementation ambassador had training, a changed mindset, started addressing more LVHBNC, saving minutes and got guidance on how to reduce care	- High work pressure; reviewing and addressing clients one-by-one takes a lot of time - De-implementation ambassadors kept the process mainly to themselves
De-implementation between professionals	- Compression stocking consultation; led by OTs, if clients cannot attend the consultation and OTs can come to their home	- Still need good collaboration with GPs - If you say no, GPs go to other healthcare organizations - GPs continue to request and promise LVHBNC to clients and relatives
De-implementation on care aids and devices	- Care aids and devices available in the office and practicing together with the team, or to borrow and practice by clients to speed up the process	- Reimbursed only if devices or care aid is requested by OTs
External factors on the de-implementation	- Decrease in care requests from GPs for compression stockings was observed, no new care requests in which client could do it themselves with a care aid - New care requests for bandaging are immediately referred to the compression stockings consultation for care after bandaging - New bandage products are experienced more comfortably for the client and much less extra phone calls to come and redo them in between prescribed time - Experience more time and space, allowing them to be put into other care, take on new clients and no need to reject clients - Decrease in specific LVHBNC care during routes, decrease of frequencies in visiting clients, and decrease in minutes per client - More opportunities for providing care that matches the level of education, but also more complex and specialist care	- Still high work pressure, but just different care - To guide clients, you need more time first - Care will still be provided by other district nurses and teams - Delay in scheduling an appointment for compression stocking consultation - Clients had an appointment with compression stocking consultation they practiced once with the care aid, it was ordered but care aids have long delivery time. Clients have not practiced again, and homecare professionals do not know how the care aid works, with the result that clients have become demotivated from using it
Maintenance in the work care process	- Conducting need assessments; be alert and aware in taking needs assessments of why client’s needs care, what client can do himself, what can environment do and what care do we provide, agreements are made in advance and listed in care plans to evaluate on - Compression stockings consultation; Two teams use a new work process for clients with compression stockings. OTs receive these care requests, evaluate which care aid is fitting and these clients no longer need care - Appropriate bandaging material is increasingly prescribed by GPs - Include the topic LVHBNC in the training for registered nurses level 6	
Maintenance by de-implementation ambassadors	- Reduce one LVHBNC practice completed; Now looking further to seeing which actions are still LVHBNC and can be replaced by a device - Some teams have reviewed all current clients for LVHBNC and now it is important to remain alert about this when taking on new clients. Other teams are still reviewing current clients, but already implementing new ways of conducting needs assessments - Once a month meeting with care contacts for the clients to discuss goals, how is the client doing, their changes in care and discuss the topic LVHBNC - Further disseminate within the organization with help from management	

*LVHBNC* Low-value home-based nursing care, *GPs* General practitioners, *OTs* Occupational therapists

*“We have 15 different GPs in the district […] so it is very difficult. I do not think the collaboration here is always ideal, but that is simply because too many health care professionals/GPs are involved.” participant 5.*

De-implementation ambassadors recognized the importance of engaging hospitals and rehabilitation centers. However, they were unable to reach these organizations during the process due to the large number of hospitals and diverse staff members operating in the area (Table 4).

*“Hospitals are large healthcare organizations and here in the region you already have several, so you don't really know who the physician is to contact. I find that quite a puzzle.” participant 2*

#### Adoption

This section described the adoption of homecare professionals’ and de-implementation ambassadors’ experiences with the role of the de-implementation ambassadors.

##### De-implementation ambassadors and homecare teams

Twelve de-implementation ambassadors completed training during phase two of the DIMPLE study (Supplementary materials 2). However, six of them dropped out later due to team reorganization (*n* = 2) or job changes without replacements (*n* = 4).

Initially twelve teams across three homecare organizations agreed to adopt the DIMPLE program. However, six teams discontinued their participation following the withdrawal of their designated de-implementation ambassadors. The remaining six teams participated in de-implementation efforts. These participating teams consisted of 7 to 14 members each and included homecare professionals with varying educational levels (see Table 5).

**Table 5:** Profession and educational levels of the participating teams

Professional and educational levels*	Team 1 (*n* = 11)	Team 2 (*n* = 8)	Team 3 (*n* = 7)	Team 4 (*n* = 13)	Team 5 (*n* = 14)	Team 6 (*n* = 9)
Registered Nurse (Level 6)	3 (27.3)	1 (12.5)	1 (14.3)	2 (15.4)	2 (14.3)	1 (11.1)
Registered Nurse (Level 4)	3 (27.3)	0	1 (14.3)	3 (23.1)	2 (14.3)	2 (22.2)
Certified Nursing assistant – individual healthcare (Level 3)	4 (36.4)	3 (37.5)	4 (57.1)	0	2 (14.3)	2 (22.2)
Certified Nursing assistant – general healthcare (Level 2 or 3)	0	0	0	7 (53.8)	0	4 (44.4)
Health and Welfare assistant (Level 2)	1 (9.0)	4 (50)	1 (14.3)	1 (7.7)	8 (57.1)	0

*Information on homecare professionals job descriptions and levels, see Supplementary materials 1

** No trainees and self-employed workers included in the team formation

All six teams successfully reduced the incidence of daily showering, bathing and/or dressing. Additionally, four teams introduced the use of a care aid to assist clients in putting on and taking off compression stockings, two teams replaced traditional bandages with self-adhesive bandages. Three teams independently implemented further reductions in low-value care, including: providing eye drops with a care aid, initiating recommended treatment for intertrigo, and introducing medication devices for client-administered medication (Fig. 1 and Table 2). No quantitative data was collected for these last three practices due to the small number of clients involved, their preventive nature, or late-stage implementation**.**

De-implementation ambassadors found it exciting to start reducing low-value care (Table 4). However, as registered nurses (level 6) with many responsibilities, they found it challenging to take on these additional tasks. They also observed that while homecare professionals identified low-value home-based nursing care, they often did not act on it and instead passed the tasks on to the de-implementation ambassador. To address this, de-implementation ambassadors recommended that the role be performed by at least two homecare professionals within the team, allowing for task-sharing (Table 4).

*“I suggest to have at least two de-implementation ambassadors, because you need the support and you can discuss with each other, you don’t have to do it alone. When I am occupied, I can still ask my colleague to perform the tasks.” participant 2.*

De-implementation ambassadors mentioned that the expectations and responsibilities associated with the role were demanding. They had to invest significant time initially to review all clients, but by focusing on reducing low-value care during needs assessments, they ultimately spent less time on care reduction (Table 4).To encourage clients and their relatives to reduce low-value care, homecare professionals highlighted the benefits of reducing care, such as fewer rotations and visits each week (Table 4). They emphasized the importance of identifying and addressing the root causes of resistance. For example, some clients feared not seeing their carers if they learned to do it themselves, or fears for complications, leading to reluctance in reducing care. Providing reassurance helped alleviate these concerns (Table 4). As one participant noted:

#### Influencing factors on the adoption of de-implementing low-value home-based nursing care

Various barriers and facilitators influencing stakeholders’ willingness to participate in the de-implementation process were identified among clients, relatives, team members, and GPs.

##### Facilitators for adoption

To encourage clients and their relatives to reduce low-value care, homecare professionals highlighted the benefits of reducing care, such as fewer rotations and visits each week (Table 4). They emphasized the importance of identifying and addressing the root causes of resistance. For example, some clients feared not seeing their carers if they learned to do it themselves, or fears for complications, leading to reluctance in reducing care. Providing reassurance helped alleviate these concerns (Table 4). As one participant noted:

*“We always say, so if it really does not work out, we can always restart the care.” participant 15*

Homecare professionals reported increased collaboration with GPs under the new approach, with GPs seeking their advice on appropriate materials to prescribe for clients (Table 4). Additionally, homecare professionals consulted OTs for advice on the use of various care aids. One homecare organization, including two teams, introduced a compression stocking consultation, where clients met with an OT to receive personalized advice on proper fitting and use of the care aid (Fig. 2). As a result, this organization observed a decrease in GP requests for stocking changes (Table 4).

*We start working together with the GPs, by including them in this initiative. The compression stocking consultation was set up here by […organization name…]. I could instruct the GPs that if you have clients who are getting compression stockings, send them to the compression stocking consultation first and then to us. […] Strangely enough we got very few requests. participant 12*

Additionally, the new self-adhesive bandages implemented by homecare professionals improved client comfort and significantly reduced extra phone calls to redo bandages between scheduled changes (Table 4). Homecare workers also reported having more opportunities to provide care aligned with their level of training. They experienced increased time and flexibility to deliver other types of care, to accept new clients, and avoid having to turn away clients (Table 4).

##### Barriers for adoption

Homecare professionals identified several client and relative attitudes that hindered the process. Some clients felt entitled to receive care, while relatives found reassurance in daily visits from homecare professionals. Others believed it was solely the homecare professionals' responsibility, and that they did not have to pay for care aids (Table 4). To address these misconceptions, a few homecare professionals found that providing clients and relatives with a breakdown of care costs compared to their health insurance payments helped clients and relatives understand the importance of reducing the care. As one participant explained:

*“Clients and relatives think it doesn't cost that much, until you show them what the costs really are. What we claim for the care per month, compared to what they pay in healthcare insurance, and then they are more open to understand the importance” participant 5.*

According to the de-implementation ambassadors, no complications such as falls, hospitalizations, or functional decline, were reported during follow-up evaluation interviews with clients to monitor progress and potential complications.

#### De-implementation

This section describes homecare professionals’ experiences with the de-implementation process, strategies they used during the process, and the identified facilitators and barriers for reducing low-value home-based nursing care practices.

##### Facilitators for de-implementation

De-implementation ambassadors found the step-by-step plan helpful in reducing low-value care and valued brainstorming and sharing results with fellow ambassadors. Additionally, coaching meetings and practicing engaging conversations were useful for performing their role effectively (Table 4). Homecare professionals observed a shift in ambassadors’ mindsets after training, noting increased commitment to addressing low-value practices and optimizing care time. However, some homecare professionals were unaware of who the de-implementation ambassador was or what their role entailed, partly due to insufficient communication about the project within the team.

*“I notice that I've kept the de-implementation process a little to myself.” participant 2*

Homecare professionals observed that clients often regained independence through the use of care aids. To guide clients and relatives, they provided visual materials and hands-on practice with care aids as part of de-implementation strategies (Fig. 2). Additionally, they facilitated involving relatives and clients in decision-making, for example, such as determining which days clients would manage care themselves and stepwise reducing care.

*“Clients are happy about regaining independence; they say, I am no longer dependent on you. I can spend my day how I want to spend it.” participant 6*

Celebrating successes played a key role in encouraging the reduction of low-value care, as did open discussions when differences in expectations about care delivery arose. One de-implementation ambassador noted that reducing care is easier to communicate and follow up when working with a small group of homecare professionals (Table 4).

*“So where care needs to be reduced there are usually only 2 or 3 professionals involved. It is easier to discuss with each other compared to if you have 10 different professionals involved.” participant 8*

Homecare professionals also found that fostering awareness within their team motivated the process. Ensuring that time was allocated for these practices helped prevent unnecessary care from limited resources for those in genuine need. Additionally, a supportive team culture – where colleagues collectively promoted and adhered to the new approach – facilitated change.

##### Barriers for the de-implementation

Homecare professionals also found that discussions about showering were more challenging than introducing care aids (Table 4).

*“Just tell someone that they're not going to be assisted with showering daily anymore and that we are going to reduce to 3 times a week. Those conversations are not very pleasant to have and in general not received very well. I have less trouble with conversations for other low-value care practices”. participant 1*

Additionally, some homecare professionals, particularly nurse assistants, found it difficult to say ‘no’ to clients and relatives, fearing resentment if they refused to provide care (Table 4). Many were also unaware that they were still performing low-value care, holding onto the outdated mindset that the ‘client is king’. As one participant noted:

*“I think it is something that we used to learn. The vision of the client as king or queen. We are here to cuddle the clients and put them at ease. I think that used to be the nurses' job”. participant 2*

Despite the increased cooperation with GPs, homecare professionals observed that when a team denied a request for care, GPs sometimes approached other organizations with the same request and that the situation was further complicated by other homecare teams continuing to accept and provide the requested services. Moreover, while a decrease in compression stocking consultations was noted, delays in scheduling appointments and receiving ordered care equipment often demotivated clients using it. To address this, homecare professionals took the initiative to assist clients with available equipment in the office. This turned an obstacle into a success factor contributing positively to the process (Table 4).

*“Sometimes I have care aids at the office, and I will use those, and I hope the client can use this aid. They will have to order an aid at home and then we can take our own care aid back to the office.” Participant 14*

Additionally, a high workload posed a significant barrier to process of de-implementation of low-value care. With other pressing demands, reducing care became a lower priority among homecare professionals. As a result, agreements were not always kept, and professionals increasingly took over care tasks (Table 4).

#### Maintenance

Following the implementation of care reduction strategies, structural changes in work processes were observed and discussed. Additionally, future aspects were discussed.

##### Structural changes in work care processes

Some teams have made their practice of reducing low-value care of selected interventions the standard of care and are now reviewing current interventions to identify opportunities for reduction. Other teams have reviewed all current clients and emphasized the importance of remaining critical when assessing new clients. Additionally, de-implementation ambassadors aim to disseminate de-implementing low-value care across organizations with the support of management and to involve homecare professionals from other teams. They also intend to incorporate the topic in the training of other registered nurses, ensuring they recognize the importance of critically reviewing care based on the value for the client (Table 4). GPs now also request the appropriate bandaging materials after being educated about the different techniques. (Table 4).

## Discussion

This study aimed to assess whether the deployment of de-implementation ambassadors, combined with the DIMPLE strategies, led to a reduction in low-value home-based nursing practices. Additionally, to gain a clear understanding of the de-implementation process and the strategies employed, we integrated the effect evaluation with a process evaluation using the RE-AIM framework [19]. Our findings indicate that the combination of de-implementation ambassadors and DIMPLE strategies resulted in a significant reduction in time spent on low-value care nursing care activities in homecare. Specifically, time savings included 130 h on activities of daily living, 54 h on the care of compression stockings, and 8 h per week on changing bandages. These findings align with a recent review on the effectiveness of de-implementation strategies to low-value care [27]. Furthermore, involving clients and relatives in decision-making and providing reassurance proved beneficial. However, homecare professionals reported challenges in refusing requests from clients and relatives, despite recognizing the importance of self-reliance. Additionally, homecare professionals noticed an increased positive commitment to reducing unnecessary care among de-implementation ambassadors. Ambassadors themselves described their role as intense, challenging, and exciting. Collaboration and involvement with other healthcare professionals like GPs, and OTs facilitated the reduction of low-value nursing care.

Champions—defined as individuals who actively promote and facilitate the adoption of innovations—have been shown to play a pivotal role in overcoming resistance, fostering stakeholder engagement, and sustaining momentum throughout the implementation process [28–31]. There are mixed results on their effectiveness and is often attributed to their ability to leverage informal influence, build trust among peers, and tailor messaging to local contexts. Hall et al. in their systematic review of guideline implementation in long-term nursing homes, found low certainty evidence that champions, when part of multicomponent interventions, may improve guideline adherence [29]. Notably, no studies were identified that evaluated the effect of nursing champions as a stand-alone strategy compared to no interventions or alternative strategies [29]. Consistent with broader implementation science literature, the impact of champions is not uniform and appears to be moderated by contextual factors such as organizational readiness, leadership support, and the complexity of the intervention [28–31]. A previous review on de-implementation strategies found that only 5% (15 of 310) of studies utilized ‘identify and prepare champions’ strategy [32]. Emerging evidence suggests that the role of champions in de-implementation may involve distinct tasks compared to their role in implementation. Unlike implementation, which often involves generating enthusiasm for adopting new practices, de-implementation requires navigating resistance to discontinuing long-standing routines. In our study, we applied this approach by introducing de-implementation ambassadors to guide the de-implementation process. Homecare professionals reported a shift in mindset and increased engagement in tackling low-value care due to the activities of de-implementation ambassadors. Our findings highlight the importance of giving these ambassadors a visible role, especially in teams where team members provide care in clients' homes and have limited visibility within their organizations. This suggests that while champions are instrumental in both implementation and de-implementation, the competencies and approaches required may differ substantially depending on the nature of the change being pursued [33]. Their role activate mechanisms of influence, such as peer modeling and trust-building, that ultimately lead to reduced low-value care. More research is needed to understand these mechanisms to effectively employ de-implementation ambassadors as champions in the field of nursing and beyond.

While homecare professionals were aware of low-value care and the need for its reduction, they relied on de-implementation ambassadors to initiate and drive de-implementation efforts. De-implementation ambassadors themselves recommended having at least two team members in this role to share responsibilities and facilitate brainstorming. This aligns with Damschroder’s findings [15], which suggest that while a single champion can implement new innovations, multiple champions are often required to enable behavior change and ensure successful implementation.

Before reducing care, homecare professionals assessed its value to clients, recognizing that its necessity varied based on individual circumstances [34, 35]. This client-centered evaluation enabled appropriate reductions. In our study, homecare professionals – being aware of clients’ conditions, their abilities and their support networks – played a key role in reviewing each client’s situation. Additionally, they actively involved clients and relatives in shared decision-making, collaborating on care reduction schedules and agreements. Previous studies have similarly shown that shared decision-making can reduce low-value care by an average of 31% [12, 36]. Engaging stakeholders through open conversations proved to be an effective strategy. However, professionals found it challenging to discontinue care without offering an alternative. This challenge aligns with existing research indicating that stopping established practices is often harder than adopting new ones, regardless of the level of supporting evidence [7].

Several unintended consequences could emerge when low-value care is reduced without sufficient consideration of client’s individual needs and experiences. First, clients may perceive such initiatives as primarily cost-driven rather than quality-oriented, potentially eroding trust in their care providers and the wider healthcare system [37, 38]. Second, the reduction or removal of care services may unintentionally shift responsibilities to informal caregivers, such as partners or family members, increasing their burden [39]. Third, clients may experience adverse outcomes, including increased risk of falls, hospital admissions or worsening of symptoms, ultimately resulting in a deterioration in health and need for additional care [37, 38]. Further, reducing care could led to decreased client adherence to therapy, which sometimes resulted in deteriorating health and requiring additional treatment time. But based on the evaluation conversations with clients no unintended adverse outcomes following reducing or stopping low-value care were reported. Beyond physical consequences, reducing low-value care in the home setting may also have emotional and relational implications. These include heightened feelings of loneliness, reduced trust in care providers or a decision to hire another homecare organization. The lack of client involvement in the current study limits our ability to fully assess these potential outcomes and highlights the importance of including client perspectives in future de-implementation research.

Collaboration and communication with other healthcare professionals were considered crucial in facilitating the process. An example was the introduction of compression stocking consultations, where OTs, homecare team, and GPs collaborated. Expanding these initiatives to other practices, such as applying eye drops with a care aid, could further reduce low-value care. Stakeholder collaboration has been recognized as a facilitator for addressing low-value care [12]. However, de-implementation ambassadors did not engage with hospitals or rehabilitation centers, as these facilities continued referring clients to homecare organizations even for low-value care. Instead, their efforts remained focused on their homecare teams. To effectively involve external healthcare organizations, management support is crucial in establishing policy agreements aimed at reducing low-value care. Such agreements could also address the barrier reported by homecare professionals: the persistence of low-value home-based nursing care within other homecare teams and organizations, which continued both performing and requesting these services. In addition to management support, nurses could teach hospitalized patients how to use equipment or encourage self-care before discharge to home.

Another strategy used by homecare professionals was engaging in conversations with clients, relatives, and other stakeholders. Similarly, in the study by Parchman, Palazzo [33], champions also engaged in conversations with stakeholders to increase support and drive behavior change. In our study, homecare professionals noted that conversations involving de-implementation without offering an alternative were particularly challenging. This aligns with findings by van Bodegom-Vos et al. [6], who compared implementation and de-implementation processes and concluded that abandoning low-value care practices is more challenging than adopting new ones, regardless of the level of supportive evidence.

### Strengths and limitations

A key strength of our study was the pragmatic approach, which included regular coaching meetings to support de-implementation ambassadors and enhance the effectiveness of care reduction. Secondly, tailored strategies were developed based on the organizational context, teams, and individual professional level, building on previous work from the DIMPLE study. While many studies primarily focus on ‘fidelity’ as a single measure of implementation success, we structured our process evaluation using the RE-AIM framework. This approach provided a more complete perspective on de-implementation.

Two limitations of our study should be acknowledged. First, data were manually collected by de-implementation ambassadors, which may have increased the likelihood of errors. Second, no clients were interviewed because there were no suitable cases at the time of the interviews. This limited the insights to the perspectives of homecare professionals alone. Including quantitative measures would have strengthened the study’s rigor. However, based on the evaluation conversations with clients no unintended consequences following reducing or stopping low-value care were reported.

### Recommendations for further research

Future research should focus on expanding and adapting the ambassador program to better align with their roles, ensuring commitment from both de-implementation ambassadors and management to support the long-term sustainability of de-implementation efforts. Next, future research should explore client- and caregiver-reported consequences of de-implementing low-value care in homecare settings. This could involve structured interviews and tailored questionnaires for both clients and informal caregivers, aimed at capturing the broader social, emotional and relational impacts of reducing low-value care. Such insights are crucial to inform the development or refinement of de-implementation strategies that are not only effective but also person-centered and responsive to the lived experiences of those most directly affected [37, 38]. Additionally, further investigation is needed to explore the sustainability of de-implementation low-value home-based nursing care, including how these efforts are disseminated within and outside homecare organization, and the potential role de-implementation ambassadors could play in this dissemination. It would also be valuable to explore the long-term effects of tailored versus standard strategies on reducing low-value care. Additionally, exploring how multiple ambassadors within a team can enhance dissemination and sustainability – both within and beyond homecare organizations – is recommended.

## Conclusion

This study demonstrates that low-value home-based nursing care can be significantly reduced or even eliminated through the deployment of de-implementation ambassadors, as part of the homecare team, and tailored strategies. As a result, some clients regained independence. Key facilitating factors included providing reassurance, using a stepwise approach to care reduction, and involving clients and their relatives in the process. While de-implementation ambassadors played a crucial role in reducing care, they found the task both challenging and intense, yet also rewarding. Further research is needed to refine strategies and scale up efforts to de-implement low-value home-based nursing care.

## Supplementary Information

Supplementary Material 1.

Supplementary Material 2.

Supplementary Material 3.

Supplementary Material 4.

Supplementary Material 5.

## Data Availability

Data is available on request from the authors.

## Abbreviations

RE-AIM Framework: Reach, Effectiveness, Adoption, Implementation, and Maintenance
GPs: General Practitioners
OTs: Occupational Therapist
