# Supplementary material for: De-implementation of low-value home-based nursing care: an effect and process evaluation
Source: Implement Sci Commun. 2025 Oct 1;6:99. doi: 10.1186/s43058-025-00785-y (PMC12487625; doi:10.1186/s43058-025-00785-y)
Supplement: Supplementary file 1 — Supplementary Material 1. [file 43058_2025_785_MOESM1_ESM.pdf]

Supplementary materials 1: Homecare professionals job' descriptions in the Netherlands

Homecare professionals job' descriptions in the Netherlands*

**Profession and educational level**	**General task description**
Registered Nurse (level 6)	High complex nursing care, responsible for quality of care and improvements, and conducting needs assessment**
Registered Nurse (level 4)	Complex nursing care, creating care plans and evaluate what care is necessary
Certified Nursing assistant – individual healthcare (level 3)	Provide and assisting with low complex nursing care and creating care plans
Certified Nursing assistant – general healthcare (level 2 or 3)	Provide and assisting with personal care tasks and organizing activities for clients
Health and Welfare assistant (level 2)	Domestic and light nursing care tasks

* Verpleeg collectief particuliere thuiszorg (1)
** These assessments were held at the start of care provision and involved collaborating with clients and their networks to determine required care, with a focus on strengthen clients' ability to care for themselves and to promote, achieve and sustain the performance of necessary activities (2,3)

References

1. Verpleeg collectief particuliere thuiszorg. Opleidingen en niveaus voor werken in de Verpleging, Verzorging & Thuiszorg (VVT): Verpleeg collectief particuliere thuiszorg; [updated 2024. Available from: https://verpleegcollectief.nl/Opleidingen-en-niveaus-in-de-thuiszorg.

2. Richard AA, Shea K. Delineation of self-care and associated concepts. J Nurs Scholarsh. 2011;43(3):255-64.

3. Schwenke M, van Dorst J, Zwakhalen S, de Jong JD, Brabers AEM, Bleijenberg N. Measures to improve patient needs assessments and reduce practice variation in Dutch home care organizations. Nurs Open. 2023;10(5):3052-63.
