# Supplementary material for: De-implementation of low-value home-based nursing care: an effect and process evaluation
Source: Implement Sci Commun. 2025 Oct 1;6:99. doi: 10.1186/s43058-025-00785-y (PMC12487625; doi:10.1186/s43058-025-00785-y)
Supplement: Supplementary file 2 — Supplementary Material 2. [file 43058_2025_785_MOESM2_ESM.pdf]

Supplementary materials 2: Training program

Aim: De-implementation ambassadors understand the steps to follow in de-implementing low-value home-based nursing care, they are able to guide and support team members during the de-implementation process, and they have tools to overcome resistance in the team, by clients or other healthcare professionals.

Content of training coaching program

Preparation:

- Submission of preparation assignment

Content:

1. Information on de-implementation science
2. Reviewing the current situation in the team on the low-value home-based nursing care practices
3. Understanding which factors influence the low-value home-based nursing care practices and whether these are a barrier or facilitator for the de-implementation
4. Determine the de-implementation strategies needed and for which stakeholders they could be used and the expected action
5. Coaching session: "How do you personally handle new innovations and what attitudes can you expect from colleagues and how might you act upon these attitudes "
6. Coaching session: "Engaging in difficult conversations and motivational conversations"
7. Creating a de-implementation plan for low-value home-based nursing care in the team

After 6 months

8. Presenting results and exchange experiences with other de-implementation ambassadors

After one year

9. Presenting results and exchange experiences with other de-implementation ambassadors

Coaching meetings

Every three months the de-implementation ambassadors had a support meeting with the researcher.

They prepared the following questions prior to the meeting

- Which goals were defined, and whether these were achieved,
- The reasons of achievement or not
- Which follow-up steps they were going to undertake in order to complete the de-implementation, and
- Who they were going to need in order to achieve the de-implementation
