# Supplementary material for: De-implementation of low-value home-based nursing care: an effect and process evaluation
Source: Implement Sci Commun. 2025 Oct 1;6:99. doi: 10.1186/s43058-025-00785-y (PMC12487625; doi:10.1186/s43058-025-00785-y)
Supplement: Supplementary file 3 — Supplementary Material 3. [file 43058_2025_785_MOESM3_ESM.pdf]

Supplementary materials 3: Tailored Strategies

<table>
<tr><th>Determinants about …
(Cremers et al., 2024)</th><th>Method to change
(Kok et al., 2017)</th><th>Inventions for strategies (Powell et al., 2015)</th><th colspan="7">Operationalization of interventions
(Proctor et al., 2013)</th></tr>
<tr><td colspan="10">Clients and relatives’</td></tr>
<tr><td rowspan="6">Knowledge
(e.g. not aware of the need to change, lack of knowledge on low-value care)</td><td rowspan="6">Advance organizers</td><td rowspan="2">Involving clients and relatives

Preparing clients and relatives to be active participants

Intervene with clients and relatives to enhance uptake and adherence</td><td colspan="7">1. Engaging in conversations with client and relatives to discuss reducing low-value home-based nursing care</td></tr>
<tr><td><u>Actor</u>:
Homecare professional or de-implementation ambassador</td><td><u>Actions</u>:
- planning, preparing, conducting conversations.
- Set goals and strive to self-care
- If necessary involve other healthcare professionals (e.g. social work, occupational therapist, general practitioner)
- plan and conduct conversations to evaluate</td><td><u>Action target:</u>
Clients and relatives</td><td><u>Temporality:</u>
During conducting needs assessments or during conversations to evaluated care</td><td><u>Dose:</u>
- plan conversation to evaluate care in the first month
- plan conducting needs assessment.
- plan conversation to evaluate reduction two-three weeks after reducing</td><td><u>Implementation outcome affected</u>
Fidelity</td><td><u>Justification</u>
To address the barriers and facilitators</td></tr>
<tr><td rowspan="2">Distribute educational materials

Develop educational materials</td><td colspan="7">2. Make use or develop material, written in form of flyer/folder of visual to inform clients and relatives</td></tr>
<tr><td><u>Actor:</u>
Homecare professional or de-implementation ambassador</td><td><u>Actions:</u>
First:
- Use existing informational materials
Second:
- No materials, than develop materials (short/compact/understandable text with pictures)
- Offer materials during conversations with the client and relatives</td><td><u>Action target:</u>
client and relatives</td><td><u>Temporality:</u>
during conversation to evaluate care or during conducting needs assessments interview.</td><td><u>Dose</u>:
Ones, during conversations</td><td><u>Implementation outcome affected</u>
Fidelity
Sustainability</td><td><u>Justification</u>
To address the barriers and facilitators</td></tr>
<tr><td rowspan="2">Conducting educational meetings</td><td colspan="7">3. Conducting educational meetings for clients and relatives</td></tr>
<tr><td><u>The actor:</u>
Homecare professionals or other involved</td><td><u>Actions</u>
- plan educational meeting, inform and invite participants, determine the information needed (care aids,</td><td><u>Action target:</u>
client and relatives</td><td><u>Temporality:</u>
From the start of the de-implementation</td><td><u>Dose:</u>
Monthly, (more of less if necessary)</td><td><u>Implementation outcome affected</u>
Fidelity</td><td><u>Justification</u>
To address the barriers and facilitators</td></tr>
</table>

			healthcare professional	pro/cons, possibilities, costs), conduct educational meeting, evaluate meeting					
Attitude and behavior<br>(e.g. entitled to receive care, high expectations, demanding care)	Environmental re-evaluation	Involving clients and relatives	<u>See strategy 1</u>						
		Preparing clients and relatives to be active participants							
		Intervene with clients and relatives to enhance uptake and adherence							
Expectations<br>(e.g. received the care for longer period, expectations of care by other healthcare professionals)	Environmental re-evaluation	Involving clients and relatives	<u>See strategy 1</u>						
		Preparing clients and relatives to be active participants							
		Intervene with clients and relatives to enhance uptake and adherence							
	Cultural similarity	Identify and prepare champions	**4. Prepare and train de-implementation ambassadors**						
			<u>Actor:</u><br>Researcher (MC)	<u>Actions:</u><br>- plan educational meeting, inform and invite participants, determine the information for sessions, prepare assignments<br>(see Appendix B), train de-implementation ambassadors to coach homecare professionals in engaging (difficult) conversations	<u>Action target:</u><br>De-implementation ambassadors	<u>Temporality</u><br>June 2022 to march 2024	<u>Dose:</u><br>2022- march 2023<br>- Every six weeks<br>2023- 2024<br>- September (6 m)<br>- March (1y)	<u>Implementation outcome affected</u><br>Fidelity<br>Sustainability	<u>Justification</u><br>Previous research (Damschroder et al., 2009; Huis et al., 2013)
Social network and Social isolation	Developing new social	Promote network weaving	<u>See strategy 1</u><br>(<u>Action</u>: involving other healthcare professionals: social worker)						

(e.g. using nursing contact as social contact)	network linkages								
**Homecare teams’**									
Attitude<br>(e.g. want to offer clients something, want to take care of clients, and it faster when homecare professionals do it)	Environmental reevaluation	Conduct educational meetings<br>“to discuss pros, cons and importance of reduction”	**5. Conducting educational meetings on reducing low-value home-based nursing care**						
			<u>Actor</u>:<br>De-implementation ambassador or homecare professionals	<u>Actions:</u><br>Involve team members (colleagues), planning an informative meeting, determine information for the meeting (e.g. background, alternatives, care aids, create awareness, discuss pros and cons, opportunity for discussion and making agreements etc.) conduct the informative meeting, involve other healthcare professionals for advice or expertise, distribution of information materials, and repeat information, share experiences and share feedback on de-implementation	<u>Action target:</u><br>Homecare professionals	<u>Temporality</u>:<br>Start of de-implementation	<u>Dose</u>:<br>Monthly for six months, there after once every three months	<u>Implementation outcome affected</u><br>Fidelity<br>Sustainability	<u>Justification</u><br>To address the barriers and facilitators
		Involving the homecare team	<u>See strategy 5</u>						
		Identify and prepare champions	<u>See strategy 4</u>						
Knowledge<br>(e.g. lack of knowledge on low-value care and guidelines)	Advance organizers	Conduct educational meetings<br>“to increase knowledge”	<u>See strategy 5</u>						
		Involving the team	<u>See strategy 5</u>						
Awareness<br>(e.g. lack of sense of urgency to change)	Consciousness raising	Conduct educational meetings<br>“to create awareness, share	<u>See strategy 5</u>						

<table>
<tr><td rowspan="4"></td><td rowspan="2"></td><td>experiences, and feedback”</td><td colspan="7" rowspan="2"></td></tr>
<tr><td>Involving the team</td></tr>
<tr><td>Framing</td><td>Conduct educational meetings
“to discuss pros, cons and importance of reduction”</td><td colspan="7">See strategy 5</td></tr>
<tr><td>Environmental re-evaluation</td><td>Identify and prepare champions</td><td colspan="7">See strategy 4</td></tr>
<tr><td rowspan="3">Skills
(e.g. lack of skills to use care aids)</td><td rowspan="3">Guided practice</td><td rowspan="2">Conduct educational outreach visits</td><td colspan="7">6. Use of trained persons to provide education</td></tr>
<tr><td>Actor:
Trained persons (e.g. occupational therapist or wound specialists)</td><td>Actions:
Plan meeting to educate homecare professionals on how to use care aids or supply the right treatment, but also on prevention recommendations.</td><td>Action target:
Homecare professionals</td><td>Temporality
Start of de-implementation</td><td>Dose:
Ones, or multiple meeting to reach homecare professionals in the team and organization</td><td>Implementation outcome affected
Fidelity</td><td>Justification
To address the barriers and facilitators</td></tr>
<tr><td>Identify and prepare champions</td><td colspan="7">See strategy 4</td></tr>
<tr><td rowspan="2">Team culture
(e.g. agreements are not followed or are unknown)</td><td rowspan="2">Resistance to social pressure</td><td>Identify and prepare champions</td><td colspan="7">See strategy 4</td></tr>
<tr><td>Conduct educational meetings
“to create awareness, share experiences, and feedback”</td><td colspan="7">See strategy 5</td></tr>
</table>

		Inform local opinion leaders	**7. Inform opinion leaders in the team, influencing colleagues with adopting the de-implementation**						
			Actor:<br>De-implementation ambassadors	Actions:<br>Inform colleagues and appoint opinion leaders who will together carry this theme,<br>Tasks of opinion leaders: taking the lead in the de-implementation, more support, setting an example, keeping agreements made, addressing colleagues help organize meetings, presentations and develop materials.	Action target:<br>Colleagues of the team	Temporality<br>Start of the implementation	Dose:<br>Ones	Implementation outcome affected<br>Fidelity	Justification<br>To address the barriers and facilitators
Maintenance of the de-implementation (e.g. falling back in old patterns, habits, routine working)	Countercon ditioning	Conduct educational meetings "to create awareness, share experiences, and feedback"	See strategy 5						
		Audit and feedback	**8. Collect data over a period of time, summarize and submit to homecare professionals to monitor, evaluate and adjust behavior**						
			Actor:<br>De-implementation ambassadors	Actions:<br>Collect data, setting goals for the de-implementation, evaluate to monitor, progress, evaluate goals, discuss progress with team and create awareness, address when progress is lacking	Action target:<br>colleagues in the team and organization	Temporality<br>Start of the implementation	Dose:<br>During the de-implementation, and every 3 months	Implementation outcome affected<br>Fidelity<br>Sustainability	Justification<br>To address the barriers and facilitators
	Implement ation intentions	Purposely reexamine the implementation	**9. Monitor progress, evaluate goals and use or adjust (new) strategies**						
			Actor:<br>De-implementation ambassadors	Actions:<br>Use strategies for de-implementing, setting goals for the strategies, evaluate de-implementation to monitor, progress, evaluate goals, discuss progress with team and create awareness, address when progress is lacking	Action target:<br>colleagues in the team and organization	Temporality<br>Start of the implementation	Dose:<br>During the de-implementation, and every 3 months	Implementation outcome affected<br>Fidelity<br>Sustainability	Justification<br>To address the barriers and facilitators

<table>
<tr><td></td><td>Public commitme nts</td><td>Identify and prepare champions</td><td colspan="7"><u>See strategy 4</u></td></tr>
<tr><td colspan="10">Involved healthcare professionals</td></tr>
<tr><td rowspan="6">Knowledge from other healthcare professionals
(e.g. lack of knowledge on low-value care, prescribing low-value care)</td><td rowspan="6">Advance organizers</td><td rowspan="2">Conduct educational meetings “to increase knowledge”</td><td colspan="7">10. Educative meeting to increase knowledge on the importances of reducing low-value home-based nursing care</td></tr>
<tr><td><u>Actor:</u>
The de-implementation ambassadors</td><td><u>Actions:</u>
Plan educational meeting, determine the information needed (care aids, pro/cons, possibilities, costs),
clarify tasks for other disciplines, conduct educational meeting, evaluate applications after the meeting, possibly plan another meeting, have a one-to-one conversation, or present information in a different ways</td><td><u>Action target:</u>
General practitioners and medical specialists</td><td><u>Temporality</u>
start of de-implementation</td><td><u>Dose:</u>
One-time Feb/March '23, evaluate after 3 months and assess if repeating is needed</td><td><u>Implementation outcome affected</u>
Fidelity
Sustainability</td><td><u>Justification</u>
To address the barriers and facilitators</td></tr>
<tr><td rowspan="2">Involving other healthcare professionals</td><td colspan="7">11. Engaging in conversations with other healthcare professionals to discuss reducing low-value home-based nursing care</td></tr>
<tr><td><u>Actor:</u>
The de-implementation ambassadors</td><td><u>Actions:</u>
- Prepare conversation for absent GPs and other medical disciplines (if they bring in many clients)
- Prepare evaluation meeting for GPs and other medical disciplines who continue to request current care and set expectations
- Schedule conversation
- Conduct conversation
- Evaluate whether requests are decreasing, otherwise schedule evaluation call.</td><td><u>Action target:</u>
General practitioners, medical specialists, nurse specialists, practice support workers, occupational therapists and transfer nurse</td><td><u>Temporality</u>
Start of de-implementation</td><td><u>Dose:</u>
Once Feb/March, evaluate after 3 months and assess whether evaluation call is needed</td><td><u>Implementation outcome affected:</u>
Fidelity
Sustainability</td><td><u>Justification</u>
To address the barriers and facilitators</td></tr>
<tr><td rowspan="2">Distribute educational materials</td><td colspan="7">12. Develop and/or make use of materials, written in the form of a flyer/folder or visual materials to inform other healthcare professionals</td></tr>
<tr><td><u>Actor:</u></td><td><u>Actions:</u></td><td><u>Action target:</u></td><td></td><td><u>Dose:</u></td><td></td><td><u>Justification</u></td></tr>
</table>

		Develop educational materials	De-implementation ambassadors or homecare professionals	Explaining which alternatives are available, how often care is provided and when, use existing materials to illustrate, develop pocketbook or card for other healthcare professionals. Development: short/compact/understandable text with pictures	General practitioners and medical specialists	Temporality<br>General practitioners and other medical disciplines	After a one-on-one or educational meeting	Implementation outcome affected<br>Fidelity<br>Sustainability	To address the barriers and facilitators
Collaborating between different healthcare professionals<br>(e.g. tackling low-value care together)	Enhancing network linkages	Promote network weaving	See strategy 11.						
**Homecare organisations**									
Support from the healthcare organization<br>(e.g. stimulating projects to promote self-care, supporting de-implementation)	Enhancing network linkages	Mandate change	**13. Involving the homecare organization with the de-implementation of low-value home- based nursing care**						
			Actor:<br>De-implementation ambassador	Actions:<br>- Share information/results of the de-implementation with management, other relevant people within the organization	Action target:<br>Homecare organization	Temporality:<br>Start and during the de-implementation	Dose:<br>Every three months	Implementation outcome affected<br>Fidelity<br>Sustainability	Justification<br>To address the barriers and facilitators
Spreading information within the homecare organization<br>(e.g. multiple different ways to spread information)	Behavioral journalism	Distribute educational materials in person, by mail and/or electronically	**14. Distribute educational materials (including guidelines, manuals, and toolkits) in person, by mail, and/or electronically**						
			The actor:<br>the de-implementation ambassador	Actions:<br>**Start of the de-implementation:**<br>Inform the team of the de-implementation on a platform or by mail, provide information on low-value care, on the strategies<br>**During de-implementation:**	Action target:<br>Colleagues in the team and management<br>.	Temporality:<br>Start of de-implementation	Dose:<br>Monthly	Implementation outcome affected<br>Fidelity<br>Sustainability	Justification<br>To address the barriers and facilitators

				During evaluation meetings inform the participant on progress, share and celebrate milestones					
Make agreements on the inventory of care aids, and materials (e.g. clients cannot or will not pay for care aids, opportunities to practice with care aids before purchasing care aids themselves)	Advocacy and lobbying	Develop resource sharing agreements	**15. Ordering inventory of materials, devices or care aids**						
			<u>Actor:</u> Procurement department with help from de-implementation ambassadors (and approval of management)	<u>Actions</u>: Purchasing new inventory, announcing the inventory to the homecare professionals, inform clients and use the inventory, report back to management, and evaluate	<u>Action target:</u> Procurement, Management, and homecare professionals	<u>Temporality:</u> Start of the de-implementation	<u>Dose:</u> Ones	<u>Implementation outcome affected</u> Fidelity Sustainability	<u>Justification</u> To address the barriers and facilitators
	Enhancing network linkages	Mandate change	<u>See strategy 15</u>						

Cremers, M., Wendt, B., Huisman-de Waal, G., van Bodegom-Vos, L., van Dulmen, S. A., Schipper, E., van Dijk, M., & Ista, E. (2024). Barriers and facilitators for reducing low-value home-based nursing care: A qualitative exploratory study among homecare professionals. *J Adv Nurs*.

Damschroder, L. J., Banaszak-Holl, J., Kowalski, C. P., Forman, J., Saint, S., & Krein, S. L. (2009). The role of the champion in infection prevention: results from a multisite qualitative study. *Qual Saf Health Care*, *18*(6), 434-440.

Huis, A., Schoonhoven, L., Grol, R., Donders, R., Hulscher, M., & van Achterberg, T. (2013). Impact of a team and leaders-directed strategy to improve nurses' adherence to hand hygiene guidelines: a cluster randomised trial. *Int J Nurs Stud*, *50*(4), 464-474.

Kok, G., Peters, L. W. H., & Ruiter, R. A. C. (2017). Planning theory- and evidence-based behavior change interventions: a conceptual review of the intervention mapping protocol. *Psicol Reflex Crit*, *30*(1), 19.

Powell, B. J., Waltz, T. J., Chinman, M. J., Damschroder, L. J., Smith, J. L., Matthieu, M. M., Proctor, E. K., & Kirchner, J. E. (2015). A refined compilation of implementation strategies: results from the Expert Recommendations for Implementing Change (ERIC) project. *Implement Sci*, *10*, 21.

Proctor, E. K., Powell, B. J., & McMillen, J. C. (2013). Implementation strategies: recommendations for specifying and reporting. *Implement Sci*, *8*, 139.
