# Supplementary material for: De-implementation of low-value home-based nursing care: an effect and process evaluation
Source: Implement Sci Commun. 2025 Oct 1;6:99. doi: 10.1186/s43058-025-00785-y (PMC12487625; doi:10.1186/s43058-025-00785-y)
Supplement: Supplementary file 4 — Supplementary Material 4. [file 43058_2025_785_MOESM4_ESM.pdf]

Supplementary materials 4 : Interview guide

Topic		
Introduction of the study	-	**Thanking for participating in the study**
	-	**Introduction of the interviewer**
	-	**Aim of the study**
	-	**Expected length of interview**
	-	**Questions in general or in follow-up to information letter**
	-	**Explaining that the data are processed anonymously**
Collecting Demographic data	-	**Filling in demographic survey**
Introduction	-	**Recording interview**
Openings question	-	**Your team has been reducing low-value home-based nursing care in the past nine months. How did you been experiencing this reduction?**
Reach<br>*Who participated or not and why?*	-	**Participation de-implementation ambassadors, clients, relatives, management and other healthcare professionals.**
	-	What makes you want to participate as a de-implementation ambassador?
	-	**Reaching team members, clients, relatives, management and other homecare professionals**
	-	Who did you approach to participate in reducing low-value care? How did you reach these individuals and why were these individuals important to approach?
	-	What was facilitating you to reach others (team members, management, homecare professionals and other professionals) in reducing low-value care?
	-	**Differences in characteristics of clients and relatives to be open to reduction**
	-	What factors make clients and relatives participate or not?
	-	What are the differences in characteristics between clients who are open or not
	-	**Obstacles to reach and what actions can be taken to get clients and relatives more involved**
	-	What can be done to involve more clients and relatives (or better) in reducing low-value care?
	-	What obstacles have you encountered in disseminating the reduction of low-value care?
Effectiveness	-	**Process outcomes for reducing low-value care**
	-	What are the most important outcomes you have seen from reducing low-value care and what actions were undertaken with clients and relatives?

Difference in results and unforeseen outcomes	- What effect do the actions have on the reduction of low-value care?<br>- What unwanted effects have you encountered from reducing low-value care?<br>- What impact have the results had on further reducing low-value care?<br>- **Effect of the strategies and the DIMPLE step-by-step plan**<br>- What effect does the process of reducing low-value care, as described in the DIMPLE step by step plan have on reducing low-value care?<br>- **Reduce effect of low-value care on clients, relatives and referrers**<br>- What effect did the strategies have on reducing low-value care for clients and homecare professionals?<br>- What effect did the strategies have on reducing low-value care on referrers (e.g. GPs, practice support workers and hospitals)?<br>- **Changes monitoring, sharing and job satisfaction**<br>- How did you monitor the effects in the use of low-value care? How have you shared these changes with you team?<br>- What effect has reducing low-value care had on your job satisfaction?
Adoption<br>*Willingness to participate with reduction of low-value care*	- **Participation of team members, monitoring and characteristics**<br>- What facilitates team members to participate or not participate in reducing low-value care?<br>- What are the differences in characteristics between those (both stakeholders and colleagues and clients and relatives) who were invited but not open to reducing low-value care?<br>- Who did eventually participated and how did you monitor this?<br>- What can be done to involve more team members and other disciplines in reducing low-value care?<br>- **Participation of other disciplines and influecing factors**<br>- What facilitates other disciplines such as GPs and occupational therapists to participate in reducing low-value care?<br>- What influencing factors work against participation for other disciplines?<br>- How do other disciplines respond to strategies used to reduce low-value care?<br>- What are external and environmental factors that have acted as barriers to reducing low-value care?<br>- **Participation of homecare organization**<br>- What facilitates the organization to participate in the DIMPLE study/ reducing low-value care?<br>- **Level of adoption of the process in reducing low-value care and the added value to work**<br>- What is the added value of reducing low-value care in your work?<br>- To what extent have you used the different components of the programme for reducing low-value care?<br>- Is there partial adoption of the process in reducing low-value care or full adoption of the process of reducing low-value care?
Implementation<br>*Understand under what circumstances consistency and*	- **Training and information provided**<br>- What did you think of the training offered prior to the process of reducing low-value care (consider duration, frequency and content)?<br>- Did you feel you received sufficient training to fulfil the role of de-implementation ambassador?

inconsistency occurs between personnel, environment, time and different parts of the program or policy implementation.	- How did you feel about the information provided on de-implementation of low-value care?<br>- **Support to reduce low-value care**<br>- How did you experience the support in reducing low-value care?<br>- How did you experience management support in reducing low-value care?<br>- **Integration in daily practices**<br>- How did you experience the incorporation of the reduction of low-value care into your current work?<br>- **Engaging in the process of reducing inappropriate care and monitoring fidelity**<br>- Can you explain to me how the process of reducing low-value care is performed, what strategies (actions) were used, who were involved and when?<br>- Who delivered the strategies used and did they have sufficient skills and time?<br>- How did you monitor and encourage that the program was implemented as agreed?<br>- **Keeping team members involved**<br>- How did colleagues respond to the strategies to start reducing low-value care?<br>- How did you engage team members and what did you need to do to keep them involved?<br>- **Work process changes made during implementation**<br>- What adjustments have been made to the process of reducing low-value care and the strategies used to reduce low-value care?<br>- What kind of adjustments do you think are needed to make the intervention valued for multiple teams? and disseminated within your organization, what is needed for this, and do you see a role for yourself in this?<br>- **Environmental factors, obstacles and missed during implementation**<br>- What environmental factors have influenced reducing low-value care e.g. time, amount of staff, environment etc. both positive and negative?<br>- What have been the biggest obstacles to achieving the predefined goals? Did you miss anything during the process of reducing low-value care? If yes, what and why?
Maintenance. *Understanding the sustainability of the program and the reasons for this, what benefits or disadvantages are there for individuals and the organization providing the*	- **Behoud van verminderen proces met welke strategieën**<br>- Wat wordt er nu gedaan aan het behouden van de effecten en welke strategieën worden daarvoor ingezet?<br>- Wat van het proces van verminderen van niet passende zorg is stopgezet en wat gaan gewijzigd worden? En waarom?<br>- **Nodige aanpassingen binnen organisatie om de implementatie te behouden**<br>- Welke aanpassingen zijn er denkt u nog nodig binnen de organisatie om de implementatie te behouden?<br>- Is er een duidelijke manier van financiering voor het verminderen van niet passende zorg en is dit opgenomen in de organisatie waar u werkt?<br>- **Belang van verminderen niet passende zorg binnen en buiten organisatie**<br>- Wat hebben verschillende disciplines of niveaus binnen de organisatie nodig om het belang van het verminderen van niet passende zorg in te zien/ in te blijven zien?

intervention decides to continue or stop this.	- **Invloed van project op lange termijn**<br>- Heeft het project invloed gehad voor de lange termijn voor u<br>- **Maintaining the reduction with strategies**<br>- What changes are made to maintain impact and what strategies are being used to do so?<br>- What of the process of reducing low-value care has been stopped and changed? And why?<br>- **Necessary adjustments within organization to maintain reduction**<br>- What adjustments do you think are still needed within the organization to maintain reduction?<br>- Is there a clear way of funding for reducing inappropriate care and is this included in the organization where you work?<br>- **Importance of reducing low-value care within and externa to the organization**<br>- What different disciplines or levels within the organization need to see/continue to see the importance of reducing low-value care?<br>- **Long-term impact of project**<br>- Has the project had any long-term impact for you
Conclusion	- **Did we forget to ask something?**<br>- Do you have any topics that have not been discussed but are important for this study?<br>- Do you have any questions or comments for me?<br>- **Explaining the member check**<br>- You will receive a summary of the interview, check if the summary is correct? And you can possibly add to it or suggest changes?<br>- Iets vergeten?
